# Imbalance of Endogenous Hydrogen Sulfide and Homocysteine in Chronic Obstructive Pulmonary Disease Combined with Cardiovascular Disease

**DOI:** 10.3389/fphar.2017.00624

**Published:** 2017-09-12

**Authors:** Yanjing He, Shaoyu Liu, Zhe Zhang, Chengcheng Liao, Fan Lin, Wanzhen Yao, Yahong Chen

**Affiliations:** ^1^Department of Pulmonary and Critical Care Medicine, Peking University Third Hospital Beijing, China; ^2^Department of Laboratory Medicine, The First Hospital of Sanming Affiliated to Fujian Medical University Sanming, China; ^3^Department of Cardiac Surgery, Peking University Third Hospital Beijing, China

**Keywords:** chronic obstructive pulmonary disease, cardiovascular disease, hydrogen sulfide, homocysteine, quality of life

## Abstract

**Background:** Considerable studies showed associations between chronic obstructive pulmonary disease (COPD) and cardiovascular disease (CVD), we evaluated the role of endogenous hydrogen sulfide (H_2_S)/homocysteine (Hcy) in patients with COPD combined with CVD.

**Methods:** Fifty one stable patients with COPD were enrolled (25 COPD, 26 COPD + CVD). Lung function, sputum, peripheral blood samples, serum H_2_S, Hcy, high-sensitivity C-reactive protein (hs-CRP) and tumor necrosis factor-α (TNF-α) levels were measured. Dyspnea, symptoms and quality of life were quantified by modified Medical Research Council dyspnea scale (mMRC), COPD assessment test (CAT) and St. George’s Respiratory Questionnaire (SGRQ).

**Results:** Compared with COPD group, waist circumference and body mass index (BMI) were higher in COPD + CVD group, mMRC, CAT and activity scores were also higher, high density lipoprotein cholesterol (HDL-C) was lower, total cells, neutrophils (%) in sputum and serum hs-CRP level were higher, whereas macrophages (% ) in sputum was lower. H_2_S and Hcy levels from COPD + CVD group were higher than those from COPD group, but H_2_S/Hcy ratio was lower. With increasing COPD severity, H_2_S level was decreased, however, Hcy level was increased. H_2_S level was positively correlated with FEV_1_/FVC, FEV_1_% predicted, lymphocytes (%) and macrophages (%) in sputum, but negatively correlated with smoking pack-years and neutrophils (%) in sputum. Hcy level was positively correlated with BMI and total cells in sputum. The ratio of H_2_S/Hcy was also positively correlated with FEV_1_/FVC, but negatively correlated with total cells in sputum.

**Conclusion:** The imbalance of H_2_S/Hcy may be involved in the pathogenesis of COPD combined with CVD and provide novel targets for therapy.

## Introduction

Chronic obstructive pulmonary disease (COPD) is one chronic inflammatory disease of the lung that is known to have systemic features, one of which is an increased risk of cardiovascular disease (CVD). There is now considerable evidence about associations between COPD and CVD. Cardiovascular events become an important cause of death in COPD. However, the mechanisms responsible for the increased risk of CVD in COPD patients are not known. Further elucidation of these mechanisms will provide novel targets for treatment of both the lung and cardiovascular complications of COPD.

Hydrogen sulfide (H_2_S) is a gas with smell of rotten eggs, it was discovered to be the third gasotransmitter after nitric oxide (NO) and carbon monoxide (CO) (Wang, 2002). The production of H_2_S in mammalian cells is attributable to three endogenous enzymes: cystathionine β-synthase (CBS), cystathionineγ-lyase (CSE) and 3-mercaptopyruvate sulfurtransferase (3-MST) (Kajimura et al., 2012). H_2_S plays an important role in respiratory diseases (Chen et al., 2005, 2011; Chung, 2014; Zhou et al., 2014; Zhang et al., 2015) and CVDs (Chen et al., 2007; Gao et al., 2015). Homocysteine (Hcy) is the main substrate to generate endogenous H_2_S in the body, high homocysteine is a risk factor in the cardiovascular system (Humphrey et al., 2008; Cioni et al., 2016; Liu et al., 2016; Luo et al., 2017). H_2_S and homocysteine are in the same metabolic pathway in the body and play a role in pathophysiological processes of diseases.

Previously we have found that serum H_2_S concentration was increased in patients with stable COPD and decreased in patients with acute exacerbation of COPD (AECOPD) (Chen et al., 2005); the imbalance of hydrogen and Hcy was shown in essential hypertensive children (Chen et al., 2007). It is not known whether the imbalance of endogenous H_2_S and Hcy is associated with CVD in patients with COPD. Therefore, this study was to explore the metabolic pathway alteration of H_2_S/Hcy in patients with COPD combined with CVD.

## Materials and Methods

### Study Design

This was a cross-sectional observational study. We collected data on demographics, smoking and medical history via interview or self-administered questionnaires. Dyspnea was quantified by application of the modified Medical Research Council Dyspnea scale (mMRC) which asked respondents to rate dyspnea on a 5-point scale from 0 (absent) to 4 (dyspnea when dressing /undressing). Symptoms were quantified by use of COPD assessment test (CAT). Quality of life in patients with COPD was evaluated by St George’s respiratory questionnaire (SGRQ). Lung function tests, sputum were performed.

### Subjects

Patients were recruited from clinics at Peking University, Third Hospital between September 2010 and March 2011. We enrolled 51 stable COPD patients. The diagnosis of COPD was made according to the criteria recommended by Global Initiative for Chronic Obstructive Lung Disease (GOLD) guideline (Rabe et al., 2007). Chronic airflow limitation was defined as forced expiratory volume in 1 s/forced vital capacity (FEV_1_/FVC) < 70% after using an inhaled bronchodilator, bronchodilator reversibility test revealed an increase in FEV_1_ < 12% and/or 200 ml below the prebronchodilator FEV_1_ after administration of 400 μg of inhaled salbutamol. Patients with stable COPD had no acute exacerbation of symptoms and upper respiratory tract infection in the 2 months preceding the study. Subjects with concomitant respiratory diseases other than COPD were excluded. COPD subjects were categorized as non-severe (stages I and II) and severe (stages III and IV) according to GOLD stages. All selected patients were divided into COPD group (25 patients) and COPD + CVD group (26 patients). CVD referred to hypertension, coronary heart disease (angina, myocardial infarction), left ventricular failure and cardiac arrhythmia. In COPD + CVD group, 11 patients got hypertension, 5 patients got coronary heart disease, 1 patient got left ventricular failure, and 9 patients got 2 or more CVDs. For each CVD, we diagnosed according to the guidelines (Roversi et al., 2016). Protocol for this study was approved by the institutional review board of Peking University, Third Hospital (approved number IRB00006761-2012029). Written informed consents were obtained from all participants.

### Pulmonary Function Test

Pulmonary function test was performed with a spirometer (Medgraphics, Elite Series DL, St. Paul, MN, United States). FEV_1_, FVC, FEV_1_/FVC, and FEV_1_% predicted were measured in all subjects.

### Serum H_2_S, Hcy, hs-CRP, TNF-α, Glucose and Lipids Levels

Venous blood samples were obtained from all subjects while fasting, serum was stored at -80°C until further analysis. Serum H_2_S concentration was measured with use of a sulfide-sensitive electrode (Model 9616, Orion Research, Beverly) according to our published literature (Chen et al., 2005). Hcy, high sensitive C-reactive protein (hs-CRP) and Tumor necrosis factor α (TNF-α) in serum were measured using commercially ELISA kits (R&D Systems, Minneapolis, MN, United States). Glucose, and lipids (T-CHO, total cholesterol; TG, triglyceride; HDL-C, high density lipoprotein cholesterol; LDL-C, low density lipoprotein cholesterol) in serum were measured using commercially reagent kits (Merit Choice, Bioengineering, Beijing; or SEKISUI.MEDICAL CO., LTD.).

### Induced Sputum Analysis

Sputum was collected and processed as previous described (Chen et al., 2005). In brief, all patients inhaled 4% hypertonic saline solution for 15–30 min to induce the sputum plugs from the lower respiratory tract, before that, all patients inhaled 200 μg of salbutamol for avoiding hypertonic saline solution-induced bronchoconstriction. Sputum plugs were incubated with 0.1% dithiothreitol until completely homogenized. After filtering and centrifuging, the cell pellet was resuspended in phosphate-buffered saline solution (PBS), then placed on slides and stained with Wright-Giemsa for differential cell counts of leukocytes.

### Statistical Analysis

Statistical analyses involved use of SPSS v17.0 (SPSS Inc., Chicago, IL, United States). Continuous variables were expressed as mean ± SD (standard deviation) for normal distribution. Categorical variables were expressed in number (percentage). For comparisons between two continuous variables, the independent two-sample test was used, for categorical variables, X^2^ test was used. Correlation analysis was performed by use of Spearman rank correlation. A two-tailed *P*-value < 0.05 was considered significant.

## Results

### Clinical Characteristics and Lung Function between Two Groups

The characteristics and lung function of subjects were shown in **Tables 1–3**. Body mass index (BMI) and waist circumference were higher in COPD + CVD group than in COPD group (**Table 1**), mMRC, CAT and activity scores were also higher (**Table 2**). The decrease of lung function was found in COPD + CVD group as compared to the COPD group (**Table 3**), but the difference was not statistically significant. So patients in COPD + CVD group had worse dyspnea, severer symptoms and worse quality of life.

**Table 1:** Demographics between two groups.

	COPD	COPD + CVD	*P*
	(*n* = 25)	(*n* = 26)	
Age, year	67.20 ± 8.58	70.38 ± 7.06	0.153
No. of male (%)	22 (88.0)	22 (84.6)	0.725
No. of smoking (%)	20 (80.0)	20 (76.9)	0.789
No. of current smokers (%)	12 (48.0)	13 (50.0)	0.886
GOLD stage III-IV, No (%)	11 (44.0)	13 (50.0)	0.668
Smoking pack-years	27.50 ± 20.71	24.22 ± 25.53	0.618
BMI (kg/m2)	21.40 ± 2.71	24.45 ± 2.63	0.000
Waist circumference (cm)	80.88 ± 5.86	88.65 ± 9.77	0.001


*COPD, chronic obstructive pulmonary disease; CVD, cardiovascular disease; GOLD, Global Initiative for Chronic Obstructive Lung Disease; BMI, body mass index*.

**Table 2:** Clinical characteristics between two groups.

	COPD	COPD + CVD	*P*
mMRC score	1.50 ± 0.91	2.35 ± 1.38	0.027
CAT score	13.50 ± 6.21	17.95 ± 6.47	0.029
SGRQ scores			
Total score	35.67 ± 16.02	45.75 ± 22.18	0.104
Symptom score	57.91 ± 17.02	65.16 ± 19.71	0.209
Activity score	46.26 ± 16.48	62.67 ± 28.06	0.031
Impact score	24.50 ± 17.87	30.40 ± 22.16	0.346


*mMRC, modified Medical Research Council Dyspnea scale; SGRQ, St. George’s Respiratory Questionnaire; CAT, COPD assessment test*.

**Table 3:** Lung function between two groups.

	COPD	COPD + CVD	*P*
FEV_1_/FVC (%)	54.48 ± 9.58	51.88 ± 11.83	0.394
FEV_1_% predicted	56.76 ± 18.62	48.92 ± 15.05	0.106


*FEV_1_, forced expiratory volume in the first second; FVC, forced vital capacity*.

### Glucose and Lipids in Blood

We determined whether the glucose or lipids in blood could be altered in COPD + CVD group. There were no significant differences in blood glucose, T-CHO, TG and LDL-C between two groups, but high density lipoprotein- cholesterol (HDL-C) was lower in COPD + CVD group (1.22 ± 0.21 mmol/L) than in COPD group(1.47 ± 0.35 mmol/L) (**Table 4**).

**Table 4:** The glucose and lipids between two groups.

	COPD	COPD + CVD	*P*
Glucose (mmol/L)	5.87 ± 1.36	5.50 ± 0.73	0.313
T-CHO (mmol/L)	5.20 ± 0.81	4.91 ± 0.89	0.313
TG (mmol/L)	1.51 ± 0.98	1.72 ± 0.85	0.506
HDL-C (mmol/L)	1.47 ± 0.35	1.22 ± 0.21	0.023
LDL-C (mmol/L)	3.11 ± 0.72	2.88 ± 0.58	0.307


*T-CHO, total cholesterol; TG, triglyceride; HDL-C, high density lipoprotein cholesterol; LDL-C, low density lipoprotein cholesterol*.

### Cellular Parameters in Sputum and Inflammatory Cytokines in Serum

Total cells and neutrophils (%) were higher in sputum of COPD + CVD group than those of COPD group, while macrophages (%) was lower, there was no statistical significance in lymphocytes (%) between two groups (**Table 5**). hs-CRP level in serum from COPD + CVD group (21.97 ± 9.84 ng/ml) was higher than that from COPD group (16.44 ± 5.98 ng/ml), but there was no significant difference in TNF-α level (**Table 5**). It indicated that the airway inflammation was more obvious in COPD + CVD patients.

**Table 5:** The differentiated cell proportion in sputum and inflammatory cytokines in serum between two groups.

	COPD	COPD + CVD	*P*
Total cells ( × 10^4^/ml)	184.94 ± 84.37	241.83 ± 72.34	0.037
Neutrophils (%)	68.00 ± 14.13	77.11 ± 10.82	0.037
Lymphocytes (%)	10.13 ± 5.3	8.6 ± 5.18	0.406
Macrophages (%)	21.50 ± 11.24	14.19 ± 5.59	0.019
hs-CRP (ng/ml)	16.44 ± 5.98	21.97 ± 9.84	0.025
TNF-α (pg/ml)	7.20 ± 3.52	7.30 ± 5.67	0.950


*hs-CRP, high sensitive C-reactive protein; TNF-α, Tumor necrosis factor -α*.

### H_2_S, Hcy Levels in Serum and H_2_S/Hcy Ratio

**FIGURE 1:**
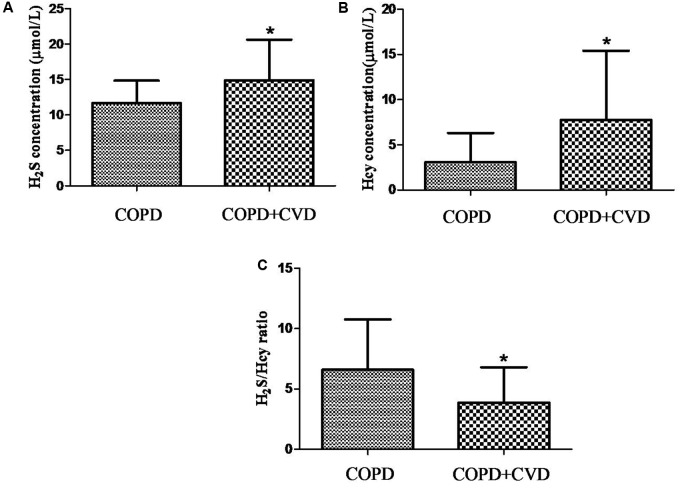
The serum H_2_S, Hcy and H_2_S/Hcy ratio in COPD and COPD + CVD groups. **(A)** Serum H_2_S concentration in both groups; **(B)** Serum Hcy concentration in both groups, **(C)** The ratio of H_2_S/Hcy in both groups. The data are described as mean ± SD. ^∗^*P* < 0.05 versus COPD.

**FIGURE 2:**
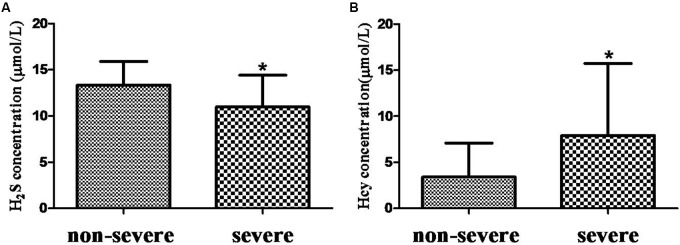
The H_2_S and Hcy in non-severe and severe groups. **(A)** Serum H_2_S concentration in both groups; **(B)** Serum Hcy concentration in both groups. The data are described as mean ± SD. ^∗^*P* < 0.05 versus COPD.

H_2_S level in serum from COPD + CVD group (14.91 ± 5.72 μmol/L) was higher than that from COPD group (11.68 ± 3.14 μmol/L) (**Figure 1A**), and it was decreased significantly with increasing COPD severity (**Figure 2A**); Hcy level from COPD + CVD group (7.76 ± 7.64 μmol/L) was higher than that from COPD group (3.08 ± 3.21 μmol/L) (**Figure 1B**), and it was increased significantly with increasing COPD severity (**Figure 2B**). We also measured the H_2_S/Hcy ratio and found that H_2_S/Hcy ratio in serum from COPD + CVD group was lower than that from COPD group (**Figure 1C**).

### Correlations among H_2_S, Hcy, H_2_S/Hcy Ratio and Other Variables

**Table 6** showed the correlations among serum H_2_S, Hcy levels, H_2_S/Hcy ratio and other variables in all subjects. Serum H_2_S level was positively correlated with FEV_1_/FVC, FEV_1_% predicted, lymphocytes (%) and macrophages (%) in sputum, while it was negatively correlated with smoking pack-years and neutrophils (%) in sputum. Serum Hcy level was positively correlated with BMI and total cells in sputum. H_2_S/Hcy ratio was positively correlated with FEV_1_/FVC.

**Table 6:** The correlations among H_2_S, Hcy, H_2_S/Hcy ratio and other variables.

	H_2_S		Hcy		H_2_S/Hcy	
	*r*_s_	*P*	*r*_s_	*P*	*r*_s_	*P*
Smoking pack year	-0.366	0.011	-0.029	0.202	0.070	0.691
BMI	0.187	0.207	0.318	0.048	-0.313	0.067
FEV_1_/FVC	0.338	0.020	-0.280	0.084	0.354	0.037
FEV1% predicted	0.306	0.036	-0.200	0.222	0.253	0.143
Cells in sputum						
Total cells	0.058	0.739	0.389	0.034	-0.392	0.035
Neutrophils %	-0.369	0.029	0.096	0.612	-0.130	0.503
Lymphocytes %	0.502	0.002	-0.081	0.672	0.167	0.385
Macrophages %	0.340	0.045	-0.151	0.425	0.082	0.672


## Discussion

In our study, we have identified the imbalance of endogenous H_2_S and Hcy in COPD + CVD. Compared with patients with COPD, patients with COPD + CVD had severer dyspnea, worse symptoms and quality of life, the ratio of endogenous H_2_S/Hcy was lower, they also had obvious local and systemic inflammation reaction.

Chronic obstructive pulmonary disease is an inflammatory disease, there is growing evidence that the inflammatory state associated with COPD is not only confined to the lung but also to the circulation system and non-pulmonary organs. Epidemiological studies indicate that COPD is associated with high frequencies of coronary artery disease, congestive heart failure and cardiac arrhythmia, independent of shared risk factors (Roversi et al., 2016). Whether they have distinct clinical characteristics is not clear.

In our study, we found that COPD + CVD patients had higher waist circumference and BMI values. BMI is an indicator reflecting the overall obesity; Waist circumference, which reflects abdominal fat content, is an indicator of abdominal obesity. Studies have shown that visceral fat produced more angiotensin, interleukin-6 and plasminogen activator inhibitor than subcutaneous fat did, (Wajchenberg et al., 2001) it had closer correlation with hypertension, diabetes, metabolic syndrome, (Liu et al., 2010) and was more important than BMI as a cardiovascular risk. So higher waist circumference is an obvious risk factor for COPD + CVD patients.

We also found patients with COPD and cardiac comorbidities had severer dyspnea, worse symptoms and quality of life. SGRQ is the most widely used questionnaire in COPD to assess the quality of life, the SGRQ score is related to the prognosis of COPD and considered as an independent risk factor for mortality (Fan et al., 2002). mMRC is a commonly used questionnaire to evaluate dyspnea of patients. CAT is a new test questionnaire which covers all aspects such as symptoms, activity and sleep, psychological and social impacts. Induced sputum analysis showed COPD + CVD patients had higher total cells, neutrophils (%) and lower macrophages (%) in sputum, which indicated that the airway inflammation was more obvious in COPD + CVD patients. These results showed that COPD + CVD patients had obvious clinical characteristics and airway inflammation.

Researchers have proposed multiple mechanisms that link coronary heart disease with COPD. Possible pathways include associations with chronic low-grade systemic inflammation, oxidative stress and shared risk factors, such as older age, cigarette smoking and environment pollution. H_2_S is found to be a protective factor in CVDs, (Yang et al., 2008; Calvert et al., 2009; Lu et al., 2017) while high Hcy is a risk factor in cardiovascular system (Humphrey et al., 2008; Cioni et al., 2016; Liu et al., 2016; Luo et al., 2017). We speculate that the imbalance of endogenous H_2_S/Hcy metabolic pathway plays an important role in the pathogenesis of COPD + CVD. It was reported that the plasma H_2_S/Hcy ratio in children with hypertension was lower than that of control (Chen et al., 2007). Plasma H_2_S level was significantly lower in patients with acute coronary syndrome than patients with stable angina pectoris or non-coronary artery disease (Gao et al., 2015). We found that serum H_2_S level was significantly increased in COPD + CVD patients and decreased with the severity of COPD. H_2_S level was positively correlated with FEV_1_, FEV_1_/FVC, and FEV_1_% predicted, which was consistent with our previous findings (Chen et al., 2005). It was shown that plasma homocysteine was elevated in COPD patients compared to healthy controls, (Seemungal et al., 2007; Fimognari et al., 2009) and elevated with COPD severity (Seemungal et al., 2007). In this study, serum Hcy level was elevated in patients with COPD + CVD and also increased with severity of COPD, which was consistent with previous findings, (Seemungal et al., 2007), however, we found that the ratio of H_2_S/Hcy in COPD + CVD patients was lower, and it was positively correlated with FEV_1_/FVC. These results suggested that the imbalance of H_2_S/Hcy may be a risk factor for COPD + CVD patients. In COPD + CVD patients, whether protein expression or activity of CBS/CSE decreasing need to be explored further.

On the other hand, many studies have shown that H_2_S played a protective role through anti-inflammation and/or antioxidant stress, for example, exerting protective effects in rats of pulmonary fibrosis or acute lung injury (Cao et al., 2014; Zhou et al., 2014; Tang et al., 2017). In contrast, high Hcy triggered an inflammatory reaction in vascular muscle cells by promoting CRP production, (Pang et al., 2014) induced inflammatory injury in endothelial cells (Han et al., 2015). In COPD + CVD patients, there was higher serum hs-CRP, total cells and neutrophils% in sputum, this indicated more obvious inflammation reaction. Both H_2_S and Hcy levels were increased in patients with COPD and cardiac comorbidities, but H_2_S/Hcy ratio was lower, maybe the higher level of H_2_S is a consequence of increased Hcy, it is a kind of compensation reaction, however, the increased H_2_S was not in proportion to the increased Hcy, the beneficial compensation of H_2_S in body may not inhibit the adverse effect of increased Hcy.

This study also showed that HDL-C was lower in COPD + CVD patients. Hcy level was positively correlated with BMI, which was consistent with the reports of and Ikkruthi et al. (2013) and Yakub et al. (2014) Hcy is also associated with lipid metabolism. In a cohort survey based in Chinese population of a community, it showed that hyperhomocysteinemia was related to increasing risk of low HDL-C and high TG, it predicted that Hcy levels might influence lipid metabolism (Momin et al., 2017). Dyslipidemia and overweight are recognized risk factors for CVD. The associations among H_2_S/Hcy, dyslipidemia and overweight may provide other targets for treatments in COPD + CVD patients.

In addition, our previous study showed that smoking can cause the decline of serum H_2_S level, (Chen et al., 2005) current study showed that serum H_2_S was negatively correlated with smoking pack-years. It is well known that smoking is a common risk factor for COPD and CVD, leading to oxidative/antioxidant imbalance and systemic inflammation. Decreased H_2_S level may reduce its anti-inflammatory and anti-oxidation capability, resulting in an increased risk for COPD and CVD.

However, our study had some limitations. First, it was a cross-sectional study, without the possibility to properly infer causality. Therefore, we need prospective studies to investigate the role of H_2_S/Hcy in metabolic pathway of COPD + CVD patients for providing some directions in diagnosis and treatment. Second, this study was a pilot study, the samples of each group were a little small, and study subjects were restricted to stable COPD patients without acute exacerbation, so our results cannot be generalized. Last but not least, We didn’t include healthy controls in this study, but in our previous study, (Chen et al., 2005) we have observed the difference of plasma H_2_S between COPD patients and healthy controls; Also, in other studies, (Seemungal et al., 2007; Fimognari et al., 2009) the difference of Hcy between COPD patients and healthy controls was observed. The objects of this study were to explore the differences of serum H_2_S and Hcy between COPD patients and COPD + CVD patients. Nevertheless, it is a foundation for future large cohort study.

In summary, a plethora of factors may be associated with the increased risks of CVD in COPD. The role of H_2_S/Hcy metabolic pathway and its clinical significance need further exploration. Future exploration of these mechanisms shall provide novel targets for treatment of cardiovascular complications in COPD.

## Author Contributions

YH, SL, YC, ZZ, and WY contributed to study concept and design. YH, SL, CL, and FL contributed to data collection. YH and SL contributed to data analysis, interpretation, and drafting. YC, ZZ, and WY contributed to manuscript revise. All authors contributed to the final approval of this manuscript.

## Conflict of Interest Statement

The authors declare that the research was conducted in the absence of any commercial or financial relationships that could be construed as a potential conflict of interest.
